# Kidney metabolism and acid–base control: back to the basics

**DOI:** 10.1007/s00424-022-02696-6

**Published:** 2022-05-05

**Authors:** Pedro Henrique Imenez Silva, Nilufar Mohebbi

**Affiliations:** 1 Institute of Physiology, University of Zurich, Winterthurerstrasse 190, CH-8057 Zurich, Switzerland; 2 National Center of Competence in Research NCCR Kidney.CH, Zurich, Switzerland; 3 Praxis Und Dialysezentrum Zurich, Zurich, Switzerland

## Abstract

Kidneys are central in the regulation of multiple physiological functions, such as removal of metabolic wastes and toxins, maintenance of electrolyte and fluid balance, and control of pH homeostasis. In addition, kidneys participate in systemic gluconeogenesis and in the production or activation of hormones. Acid–base conditions influence all these functions concomitantly. Healthy kidneys properly coordinate a series of physiological responses in the face of acute and chronic acid–base disorders. However, injured kidneys have a reduced capacity to adapt to such challenges. Chronic kidney disease patients are an example of individuals typically exposed to chronic and progressive metabolic acidosis. Their organisms undergo a series of alterations that brake large detrimental changes in the homeostasis of several parameters, but these alterations may also operate as further drivers of kidney damage. Acid–base disorders lead not only to changes in mechanisms involved in acid–base balance maintenance, but they also affect multiple other mechanisms tightly wired to it. In this review article, we explore the basic renal activities involved in the maintenance of acid–base balance and show how they are interconnected to cell energy metabolism and other important intracellular activities. These intertwined relationships have been investigated for more than a century, but a modern conceptual organization of these events is lacking. We propose that pH homeostasis indissociably interacts with central pathways that drive progression of chronic kidney disease, such as inflammation and metabolism, independent of etiology.

## Introduction

The concentration of H^+^ in biological fluids influences a multitude of biological activities in living beings belonging to all life domains. Protons are central to the understanding of life because they interact with multiple biological functions and structures. H^+^ is here a simplified notation of the actual chemical structure of the aqueous proton (whether H_13_O_6_^+^, H_5_O_2_^+^, or H_9_O_4_^+^, this is a debate beyond the topic of this article and covered by others [78, 103]). Proton concentration, most often represented in its logarithmic form, pH, determines the activity of enzymes, bioavailability of substances, protein conformation, electrostatic surface of proteins, and their capacity to interact with other proteins. Protons are so central to life that we “breathe” through them; the movement of protons through the mitochondrial or cell membrane is the mechanism by which multiple forms of life produce ATP. Regulation of pH is therefore essential for normal human physiology and is involved in pathophysiological processes. In humans, pH values can be lower than 1 in the gastric acid or above 8 in the pancreatic juice. However, blood pH is normally around 7.4, which protects organs from noxious consequences of largely altered proton concentrations. Lungs and kidneys are the main organs involved in the maintenance of pH homeostasis in humans. They achieve this task by dictating the elimination of acids and bases and together with bones supporting adequate levels of extracellular buffers. In other words, they control the balance of acids and bases. These are not recent notions given that the role of kidneys and lungs in the maintenance of acid–base balance was already recognized by Claude Bernard in the 1859s *Leçons Sur Les Propriétés Physiologiques Et Les Altérations Pathologiques Des Liquides De L’organisme* [8]. He identified that both organs transferred forms of carbonic acid between fluid compartments, which was essential to keep pH at healthy levels.

In this review, we cover in a historical perspective how kidneys contribute to acid–base balance and how disturbed acid–base conditions affect kidney health. We show that some of the most recent findings regarding the influence of pH on renal pathophysiology and metabolism relate to central topics of investigation from the end of the XIX century and most of the XX century, but with a shift in focus towards the pro-inflammatory arm of the disease, they were somehow left in the backseat in the few past decades. It is time to bring them back to the center of the debate.

## How kidneys support acid–base balance

Our understanding of how kidneys support pH homeostasis is pigeonholed through the acid–base school of thought that one follows. In one of the schools, bicarbonate is the central player in how kidneys protect the organism from acid–base disorders. This conceptual framework was derived from the Henderson-Hasselbach equation, which in turn is a product of the definition of acids and bases of Brønsted and Lowry. The other framework understands that [H^+^] is determined by the contribution of ions whose charge is unaltered at physiological pH, also known as strong ions (i.e., Stewart’s approach [114]). Here, we describe how kidneys perform their “acid–base roles” through the bicarbonate-centered framework. While most of the content reviewed in this article can be explained under the light of the strong ion approach, several of the mechanisms described here would lack parsimony. With that said, kidneys fundamentally protect pH homeostasis via reabsorption of bicarbonate and generation of new bicarbonate. These processes are briefly summarized in this section. Kidneys reabsorb almost the entire amount of filtered bicarbonate, with ~ 70–80% of it done in the proximal tubules, ~ 10–15% in the thick ascending limb of the loop of Henle, 4–6% in the distal convoluted tubule, and the remaining in the collecting duct. In every segment, it uses the same mechanism: secretion of H^+^.

When H^+^ is moved from the intracellular space to the luminal space, it reacts with a HCO_3_^−^ molecule and forms H_2_CO_3_, and in a reaction catalyzed by carbonic anhydrases, forms CO_2_ and H_2_O.

$${\mathrm{HCO}}_{3}^{-} + {\mathrm{H}}^{+}\rightleftharpoons {\mathrm{H}}_{2}{\mathrm{CO}}_{3}\rightleftharpoons {\mathrm{CO}}_{2}+{\mathrm{H}}_{2}\mathrm{O}$$

**Fig. 1:**
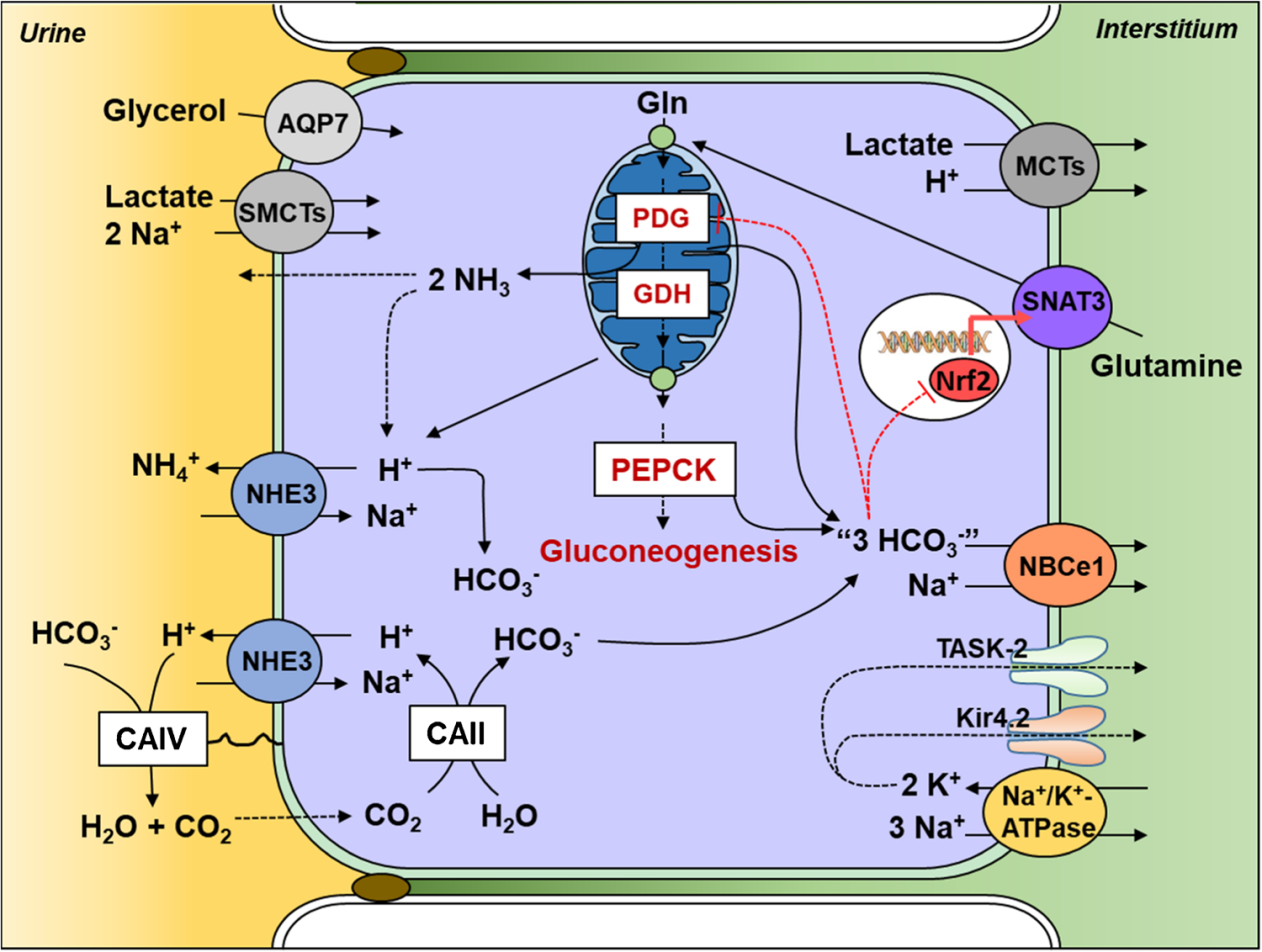
Bicarbonate reabsorption and formation of new bicarbonate via ammoniagenesis in coordination with glutamine metabolism, gluconeogenesis, and activity of potassium channels in the proximal tubule. Secretion of H^+^ via NHE3 or H + -ATPase (not shown) leads to reabsorption of HCO_3_^−^ via NBCe1 (and AE2 in the segment 3). Ammonia and HCO_3_^−^ are formed from the metabolization of glutamine in the mitochondria, which provides precursors for gluconeogenesis. Glycerol and lactate are additional substrates of gluconeogenesis, but they have a minor role in response to metabolic acidosis in healthy kidneys. The transcription factor NRF2 regulates the expression of the main importer of glutamine into proximal tubular cells during acidosis, SNAT3. Potassium channels in the basolateral membrane control membrane potential impacting NBCe1 activity and ammoniagenesis. *NHE3* (*SLC9A3*) sodium hydrogen exchanger 3, *NBCe1* (*SLC4A4*) electrogenic sodium bicarbonate cotransporter 1, *SNAT3* (*Slc38a3*) sodium-coupled neutral amino acid transporter 3, *NRF2* (*NFE2L2*) nuclear factor-erythroid factor 2-related factor 2, *TASK2* (*KCNK5*) TWIK-related acid-sensitive K( +) channel 2, *KIR4.2* (*KCNJ15*) inward rectifier K^+^ channel KIR4.2, *AQP7* aquaporin 7, *CAII and CAIV* carbonic anhydrase 2 and 4, respectively; SMCTs represent sodium-coupled monocarboxylate transporters 1 and 2 (*SLC58* and *SLC5A12*); MCTs represent different monocarboxylate transporter members, most probably *SLC16A1* and *SLC16A*; *PDG* (*GLS*) phosphate-dependent glutaminase, *GDH* (*GLUD1*) glutamate dehydrogenase, *PEPCK* (*PCK1*) phosphoenolpyruvate carboxykinase

CO_2_ enters the cell and with H_2_O forms H^+^ and HCO_3_^−^. Therefore, the secreted H + is also formed from the same reaction, the hydration of CO_2_. Altogether, for every H^+^ secreted, a HCO_3_^−^ is formed inside the cell. This bicarbonate is reabsorbed through the basolateral membrane and goes back into the bloodstream. In the proximal tubules, the movement of H^+^ is mostly achieved by the sodium hydrogen exchanger paralog 3 (NHE3) in the apical membrane, but also by H^+^ ATPase. Bicarbonate is reabsorbed mostly by the electrogenic sodium bicarbonate cotransporter 1 (NBCe1), but also by the anion exchanger 2 (AE2) in the segment 3 of the proximal tubule [19] (Fig. 1). Similar mechanisms are present all along the nephron, with changes in the protein paralogs. As an additional player, proton excretion also occurs via K^+^/H^+^ ATPase in type A intercalated cells.

Kidneys also display a bicarbonate-secreting mechanism in the collecting duct. Pendrin (*SLC26A4*), a Cl^−^/HCO_3_^−^ exchanger functioning as a bicarbonate secreting protein, was also identified in type B and in non-A non-B intercalated cells [108]. The basic mechanism is the same here, a proton moves through the basolateral membrane and a bicarbonate is secreted to the apical lumen. It has been suggested that pendrin is a key factor in the renal defense against alkalosis given that isolated cortical collecting ducts from alkali-loaded pendrin null mice cannot properly secrete bicarbonate [99]. In addition, these mice are prone to develop alkalosis under dietary sodium and potassium restriction [92]. The gastrointestinal hormone secretin stimulates cystic fibrosis transmembrane conductance regulator (CFTR) and pendrin activity. They work in concert promoting bicarbonate secretion in type B intercalated cells in the collecting duct [7]. Patients with cystic fibrosis carrying a mutation in CFTR show impaired renal excretion of bicarbonate [6]. Authors suggested that secretin would be a bicarbonate-regulating hormone and would be responsible for the elevated bicarbonate excretion after a meal, which is known as alkaline tide [7]. In addition, a role for pendrin in salt regulation has been proposed, and acid–base changes could also be secondary to changes in extracellular volume [122, 124]. Besides CFTR, pendrin may also function in concert with NDCBE1 (*SLC4A8*), a Na^+^/HCO_3_^−^/Cl^−^ transporter. Pendrin and NDCBE1 would generate net reabsorption of NaCl while limiting bicarbonate secretion [124].

The second fundamental activity is the formation of de novo or new bicarbonate, which means the restoration of bicarbonate consumed by the addition of fixed acids to the organism. This new bicarbonate is formed via two main mechanisms, ammoniagenesis and excretion of titratable acids. These mechanisms were identified in the first decades of the XX century, when Henderson recognized that excretion of ammonium and phosphates were essential for the maintenance of acid–base balance [51, 52]. Other forms of urinary acids were already recognized at that time [40], and these acids were termed titratable acids (but also received other names, such as free acids) [105, 121].

Titratable acid excretion is a simple process which is a consequence of a proton secreted binding bases other than bicarbonate. Therefore, bicarbonate is formed inside the cell without consumption of bicarbonate in the lumen. On the other hand, bicarbonate formation through ammoniagenesis requires steps that span almost the whole nephron. The formation of ammonium happens in the proximal tubule via biochemical reactions that start from glutamine. This amino acid is metabolized in the mitochondria producing alpha-ketoglutarate, which participates in the cytosolic gluconeogenesis. Mitochondrial and cytosolic steps yield together a net of two molecules of bicarbonate and ammonia per glutamine. Ammonia (NH_3_) is secreted to the tubular lumen and with H^+^ forms ammonium (NH_4_^+^) (Fig. 1). If the nephron ended after the proximal tubule, this would be the end of this story with positive formation of bicarbonate (new bicarbonate). However, if ammonium goes back to the bloodstream, it will consume bicarbonate in the liver via the urea cycle, which will deny new bicarbonate formation. Ammonium excretion does not follow the path of many other ions that travel through the tubule lumen to the ureter, but most of it is reabsorbed in the thick ascending limb and secreted back into the thin descending limb of the loop of Henle or in the collecting duct, thus partly bypassing the distal convoluted tubule. Different hypotheses have been proposed to explain why ammonium would take such an unconventional path before reaching the urine. As mentioned previously, this countercurrent multiplication of ammonium could avoid its reabsorption in the cortex [88, 112] (i.e., distal convoluted tubule and cortical collecting duct), but it was also suggested that NH4^+^ would contribute to NKCC2 activity and NaCl reabsorption in the thick ascending limb [128, 129]. Regardless of the potential reasons why these mechanisms could have been fixed, ammonium crosses the apical membrane of the thick ascending limb via NKCC2 substituting potassium in the process. It leaves the cell through the basolateral membrane via two mechanisms, NHE4 (NH_4_^+^ instead of H^+^ exchanged with Na^+^) or the coordinated transport of NH_3_ to the medullary interstitium in parallel with the movement of HCO_3_^−^ into the cell involving the electroneutral Na^+^-bicarbonate cotransporter NBCn1 [16, 87].

In the medullary interstitium, sulfatides facilitate the retention of ammonium, which passively diffuses as ammonia into the collecting duct via the RhCG and maybe also as ammonia or ammonium via RhBG [12, 23, 113]. Proton secretion by type A intercalated cells in parallel with ammonia transport traps ammonium into the lumen, increasing the probability of its excretion in the urine. Only after this journey is the production of new bicarbonate consolidated. Despite the observation that excretion of titratable acids and ammonia were essential for the maintenance of acid–base balance in the first years of the XX century, only in 1921 did Nash and Benedict demonstrate that ammonia was formed in the kidneys [83]. Subsequent decades saw a stream of studies trying to identify the metabolic origins of ammonium and how this mechanism was regulated in health and disease.

## Current views on the pathophysiology of metabolic acidosis in kidney disease

In this section, we cover common conditions of renal origin that generate metabolic acidosis and its management in the clinical setting.

### Metabolic acidosis in CKD

In CKD, metabolic acidosis occurs with declining kidney function and the subsequent fall of glomerular filtration rate (GFR) independent of the underlying kidney disease. The consequent loss of nephrons results in two important processes: globally reduced excretion of ammonia, but increased ammoniagenesis in the remaining nephrons. In addition, hemoglobin also functions as a buffer in the blood, and CKD is commonly accompanied by anemia, which might contribute to metabolic acidosis. Moreover, a series of phosphaturic mechanisms preserve titratable acid excretion in patients with CKD, but in end-stage kidney disease, reduction in its excretion contributes to the occurrence of overt metabolic acidosis [81]. Clinically, metabolic acidosis presents not only as normal anion gap metabolic acidosis but, in some patients, and especially in advanced stages, also as anion gap metabolic acidosis. However, a more recent theoretical construct includes earlier CKD stages by using the term “eubicarbonatemic metabolic acidosis,” which is defined by proton accumulation preceding the fall of serum bicarbonate levels [44]. Noteworthy, dietary acid load has a key role in determining the occurrence of eubicarbonatemic or overt metabolic acidosis in individuals with compromised kidney function [131].

Kidneys respond to metabolic acidosis by stimulating mechanisms that form new bicarbonate. Given that pH has highly pleiotropic effects, it is wise to look at the adaptive responses to metabolic acidosis and what possible effects their chronic activation could cause. Along these lines, Nath et al. proposed that accumulation of ammonium in the renal interstitium would trigger inflammation via the alternative complement pathway [85]. They identified that adding NaHCO_3_ to the diet reduced ammonium concentration in the renal vein and attenuated intratubular casts, tubular dilation, and interstitial fibrosis. One of the hallmarks of chronic kidney disease is the reduction in ammonium excretion. However, Simpson showed in 10 patients with CKD and acidosis that GFR falls proportionally more than ammonium excretion, which suggests that ammonium generation per nephron may increase in acidotic patients with CKD [104]. However, it has been demonstrated that ammonium binds sulfatides in the medullary interstitium [113]. Therefore, it is not clear how ammonium could trigger the activation of the complement system, unless intrarenal sulfatides are also reduced in CKD. Some key open questions need to be addressed in relation to the NH4^+^/alternative complement system hypothesis: (1) Are all forms of CKD marked by accumulation of NH4^+^ in the renal tissue? (2) Is this mechanism relevant both in the cortex and in the medulla? (3) Is NH4^+^ the actual molecule responsible for triggering inflammatory responses in CKD with acidosis? (4) What other immune responses beyond activation of the alternative complement pathway could be triggered by NH4^+^?

Kidneys also increase the activity of NHE3 in the proximal tubule in response to acidosis, which is assumed to be a mechanism supporting ammonium excretion via a Na^+^/NH4^+^ exchange [32]. At the same time, proton secretion in the collecting duct is increased which helps ammonia to be converted into ammonium and then be trapped into the tubular lumen. The hormones angiotensin II, aldosterone, and endothelin-1 support the increase in these mechanisms in the proximal tubule and collecting duct [135]. However, their chronic activation by acidosis leads to inflammatory processes and fibrosis. Studies in nephrectomized rats and patients with CKD support this hypothesis [132–134]. However, a recent randomized clinical trial with 45 patients with CKD designed to identify potential benefits of alkali therapy on the reduction of these hormones did not find a reduction in the levels of urinary renin, angiotensinogen, aldosterone, or endothelin-1 [17]. While urinary levels of these hormones might not reflect their intrarenal levels, further studies are necessary to evaluate the effectiveness of alkali therapy in reducing these harmful factors in CKD. In summary, activation of the alternative complement pathway by ammonium (published in 1985) and the toxic effect of hormones stimulated by acidosis (as shown in a long list of studies led by Donald Wesson and Jan Simoni and supported by many colleagues since the 1990s) have been established as the modern explanations for the deleterious effects of acidosis on CKD. They link the physiological responses of the kidneys against metabolic acidosis to inflammatory processes.

### Renal tubular acidosis

Renal tubular acidosis (RTA) is a condition in which tubular secretion of H^+^ and reabsorption of HCO_3_^−^ are impaired despite relatively normal GFR [110]. It was first described in the 1930s in pediatric patients with severe renal calcification, but only in the next decade would the condition be explained [2, 22, 71]. It was termed “renal acidosis,” a condition of “tubular insufficiency without glomerular insufficiency” [2]. Renal tubular acidosis is clinically characterized by normal anion gap metabolic acidosis with an alkaline urinary pH. Particularly in the last decades, deeper insights have been gained on genes involved in inherited forms of renal tubular acidosis. Dependent on the gene and localization of the defect, three different types of renal tubular acidosis are defined: (1) proximal RTA, (2) distal RTA, and (3) hyperkalemic RTA. By now, more than 25 genes have been identified to cause inherited RTA. Interestingly, polymorphisms may also cause some diseases that do not present typically as inherited RTA in patients with nephrocalcinosis or nephrolithiasis [123]. Moreover, there are still patients with inherited RTA where no mutation has been found yet, indicating that other genes or further mutations, for example, in noncoding regions, may be involved.

Hyperkalemia is commonly accompanied by lower net acid excretion and metabolic acidosis [36]. A recent study in mice has added some evidence on the role of hyperkalemia per se in the pathogenesis of hyperkalemic RTA [48]. Hyperkalemia causes metabolic acidosis by both reducing ammoniagenesis in the proximal tubule and impairing ammonia transport in the collecting duct [48]. Interestingly, deletion of Kir4.2 (*Kcnj15*) in mice disturbs the membrane potential of the proximal tubule basolateral membrane, which elevates intracellular pH and reduces ammoniagenesis, causing proximal RTA [11]. Regarding acquired forms of RTA, few studies have shed light on a specific autoimmune disease called Sjögren syndrome [30, 125]. This is a systemic disease that can involve the kidney by defective urinary acidification and subsequent distal RTA. Published data suggest that autoantibodies may be involved in the pathogenesis by potentially affecting acid-secreting type A intercalated cells in the distal tubule [120]. However, more studies are required to identify the respective antigens that may be targeted by the autoantibodies.

### Metabolic acidosis in kidney transplant recipients

Interestingly, metabolic acidosis occurs in kidney transplant recipients (KTRs) at higher eGFR levels when compared to patients with CKD [79]. This finding suggests that there may be transplant-specific mechanisms involved, and it is further supported by the fact that metabolic acidosis in KTRs typically presents with the features of renal tubular acidosis (RTA), such as normal anion gap metabolic acidosis, compared to high anion gap acidosis in patients with CKD [79]. Among the transplant-specific features, calcineurin inhibitors may be of great importance. Data from animal and human studies demonstrated that both cyclosporine and tacrolimus may affect tubular function including recent findings about the role of pendrin in the pathogenesis of distal RTA [4, 50, 74, 80, 127]. In addition, other elements, such as immunological factor associated with allograft rejection, donor-associated factors (graft from a deceased donor may be associated with a higher rate of metabolic acidosis) [18, 89], and dietary factors (white meat is associated with lower risk and dietary acid load is associated with a higher risk of graft failure) [100, 140], may also contribute to the development of metabolic acidosis in KTRs. In a recent study, we investigated the impact of metabolic acidosis and its therapy on molecular changes in renal biopsies of KTRs via RNA sequencing and immunofluorescence [59]. Our data demonstrated that metabolic acidosis in kidney transplant recipients is associated with changes in the renal transcriptome and protein expression of genes mostly involved in acid–base transport and cell energy metabolism (see section on “Metabolic acidosis and metabolism”). These changes were partly reconstituted by alkali therapy [59].

### Impact of alkali therapy on kidney function

Metabolic acidosis is common in patients with CKD, with an increasing prevalence in advanced stages of CKD. Starting in 2009, with the first open-label clinical trial, more than 10 studies have investigated the impact of alkali therapy on kidney function and CKD progression as well as proteinuria [55]. Although when comparing alkali therapy with placebo or no medication, the results were favoring sodium bicarbonate to slow CKD progression, the overall certainty of evidence is still low, and further studies are required.

### Alkali therapy in transplantation

Similar to CKD, metabolic acidosis is highly prevalent after kidney transplantation, with a reported prevalence of 12 to 58% [137]. Although by now many interventional trials have evidenced the beneficial effect of alkali therapy on preservation of kidney function in patients with CKD, no prospective randomized controlled trial has been published yet on the potential impact of alkali supplementation on graft function in kidney transplant recipients. However, a few retrospective analyses indicate an association of metabolic acidosis and graft survival [18, 89]. The first study was an observational multicenter analysis of 2318 Korean KTRs with metabolic acidosis (defined as tCO_2_ level < 22 mmol/l), demonstrating an association of metabolic acidosis with graft outcome and mortality [89]. A more recent study from France including 914 KTRs confirmed these data and reported low bicarbonate being predictive for allograft loss [18]. Furthermore, two observational studies from our center showed a positive correlation of serum bicarbonate with eGFR in the first year after transplantation and a significant association of serum bicarbonate with long-term graft and patient survival [136, 138]. A retrospective study from Japan with non-KTR patients with CKD concluded that venous pH altered the association between CKD and progression to kidney replacement therapy [62]. In other words, patients with low venous bicarbonate and acidemia were under higher risk of undergoing kidney replacement therapy than patients with low venous bicarbonate and normal blood pH. It is unknown whether the same modulation occurs between serum bicarbonate or tCO_2_ and graft failure/loss.

The Preserve Transplant Study is an investigator-initiated prospective randomized placebo-controlled single-blinded interventional trial investigating the effect of alkali treatment on graft function of KTRs with metabolic acidosis (defined as serum bicarbonate ≤ 22 mmol/l) over 2 years. Kidney graft recipients, ≥ 18 years of age, at least 12 months after transplantation, with an eGFR between 15 and 89 ml/min/1.73 m^2^ and bicarbonate of ≤ 22 mmol/l were randomized 1:1 to receive placebo or sodium hydrogen carbonate. The results of the study are expected in 2022 and will be essential to clarify whether alkali treatment in KTRs with metabolic acidosis may help to prolong long-term graft survival in this population.

## Metabolic acidosis and metabolism

**Fig. 2:**
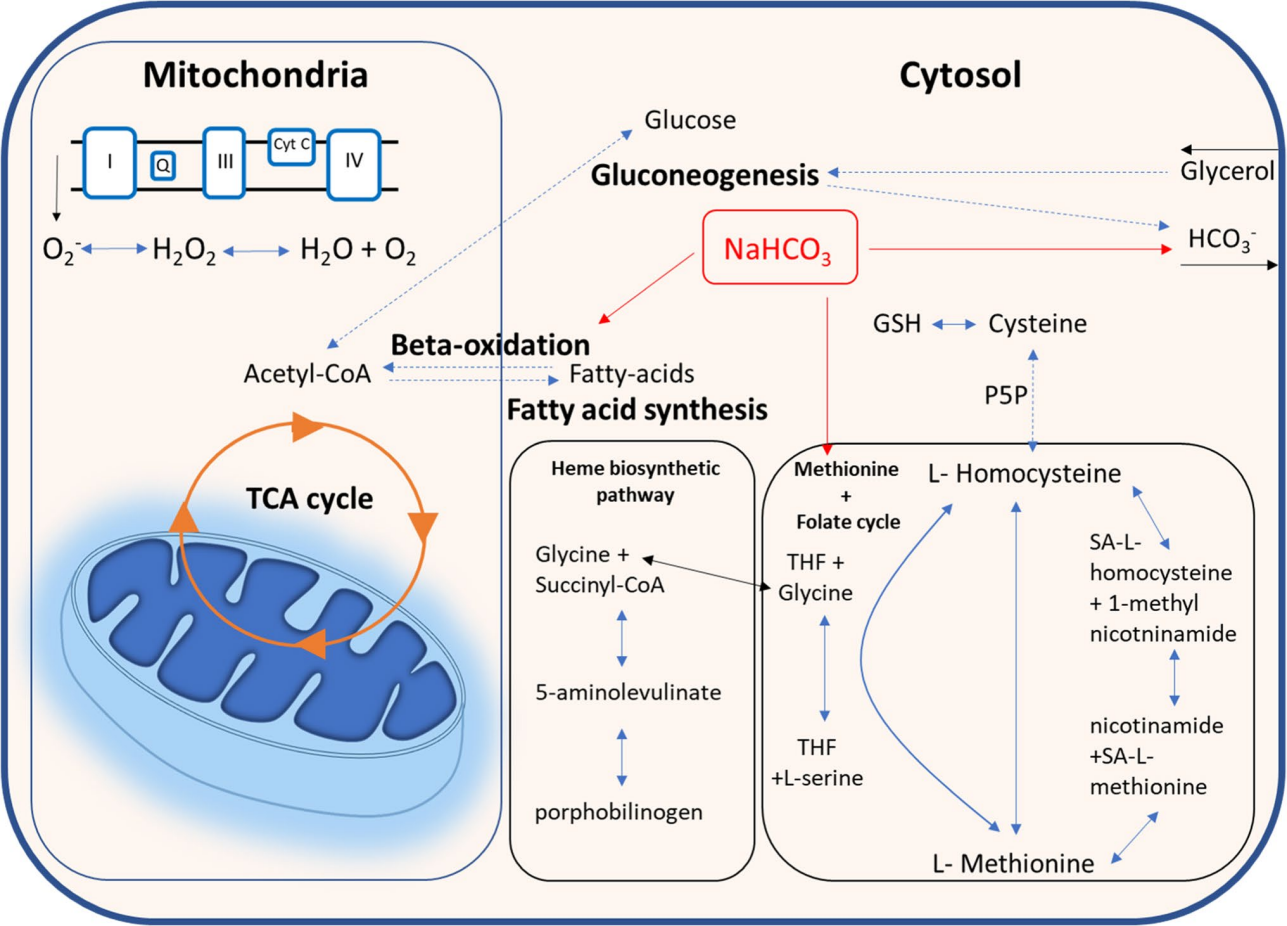
Summary of main renal metabolic pathways altered between kidney transplant recipients (KTRs) with or without acidosis. Bulk RNA sequencing data using RNA from kidney biopsies of KTRs identified genes altered between patients with or without acidosis, but with comparable eGFR. These genes participate in metabolic activities shown in this figure in black. Red lines show molecular pathways that had genes restored by alkali therapy. Blue arrows show direct biochemical reactions, and blue dashed lines show indirect biochemical reactions. Black arrows show movement of molecules. Data originally published in [59]. *TCA cycle* tricarboxylic acid cycle (also citric acid cycle or Krebs cycle), *P5P* pyridoxal-5′-phosphate, *GSH* glutathione, *THF* tetrahydrofolate

Claude Bernard observed that dietary habits from carnivores and herbivores would determine the acidity or alkalinity of the urine, therefore recognizing that what we eat imposes different challenges to the organism from an acid–base perspective [8]. The incomplete oxidation of substrates was already recognized as a source of acidification of the organism in the 1898s *Der Diabetes Mellitus*, by Bernhard Naunyn, who termed this condition as *Acidose* (acidosis) [9]. Since then and for several decades, substantial investigation on acid–base disorders focused on whole-organism metabolic studies in an attempt to identify how metabolites in humans and animal models like rabbits and dogs would respond to alkaline and acid challenges. After two decades of debate on the source of renal ammonia, glutamine was identified by Donald Van Slyke et al. in 1943 as the main fuel of ammoniagenesis in response to metabolic acidosis, a process further understood in the subsequent decades [84, 94, 106]. The close relationship between renal gluconeogenesis and ammoniagenesis (and new bicarbonate formation) ties cell energy metabolism to acid–base balance even further. Glutamine, lactate, and glycerol are the main substrates of renal gluconeogenesis, but glycerol and lactate do not participate in the stimulated ammoniagenesis in response to low pH (although lactate oxidation also yields one HCO_3_^−^) [95, 115]. During acidosis, there is a shift from the metabolization of other substrates of gluconeogenesis (e.g., lactate) towards glutamine [93]. Moreover, substrates of the TCA cycle inhibit ammoniagenesis in normal acid–base conditions, but have a less inhibitory effect during acidosis, while glycerol has almost no effects on ammoniagenesis [3]. However, patients with CKD with mildly impaired kidney function and partially preserved ammonium excretion showed almost negligible renal extraction of glutamine [119]. Another study with patients with CKD receiving oral intake of glutamine demonstrated that ammonium excretion could not be increased even under abundant availability of glutamine [130]. Authors suggested that a reduction in the enzymatic activities participating in ammoniagenesis would explain the reduced ammonium excretion. Indeed, it was later demonstrated that acute injury to the kidneys reduces the expression of ammoniagenic enzymes [68] and that both in acute kidney injury and chronic kidney disease, there is a metabolic rewiring that redirects energy metabolism away from gluconeogenesis [39]. In healthy humans and animal models, stimulation of ammoniagenesis and gluconeogenesis by acidosis increases renal glucose generation [1, 111]. Therefore, pH affects biochemical pathways that will lead to differential production and release of metabolites. The focus on cell metabolism as a key element to understand kidney disease has increased in the past recent years. Integrative approaches combining molecular and clinical data have found that metabolism and inflammation are central pathways in various forms of CKD [37, 63, 77]. Interestingly, when we subjected mice to a crystal nephropathy CKD model and treated them with oral bicarbonate, two largely restored pathways were again inflammation and metabolism [90]. Moreover, as mentioned earlier, we collected renal biopsies of kidney transplant recipients both with and without acidosis and with comparable eGFR and performed RNA sequencing. More than half of the altered genes between acidotic and non-acidotic patients were enzymes, mostly involved in cell energy metabolism activities like beta oxidation, fatty acid synthesis, interconversion between L-methionine and L-homocysteine, and others [59] (Fig. 2). We also obtained a few biopsies of patients with acidosis and treated with alkali therapy and showed restoration of genes involved in bicarbonate transport (NBCe1, pendrin, and Kir4.2), beta oxidation (ACADSB), and interconversion of L-homocysteine and L-methionine as well as glycine and L-serine (SHMT1) (Fig. 2).

Metabolic acidosis alters the redox state of mitochondrial nicotinamide adenine dinucleotide (NAD) and causes mitochondrial stress in renal proximal tubules, which affects lipid metabolism [21, 82]. Bugarski et al. have shown that oxidation of NAD by acid load injures proximal tubule cells and that alkali treatment prevents such changes [21]. Low-grade metabolic acidosis is also a necessary signal for mitochondrial remodeling in response to hypoxia. Cortical neurons exposed to pH 6.5 showed increased crista number and sustained functional efficiency under hypoxic conditions while mitochondrial fragmentation and cell death were prevented [64]. However, exposure of the same cells to more alkaline pH (6.8–7.2) or more acidic (pH 6.0) induced mitochondrial fragmentation. Renal cells are exposed to hypoxic conditions in chronic kidney disease, and similar mechanisms may operate. Also in neurons, ASIC1a mediates pH-dependent calcium transport into the mitochondria, increasing respiration and metabolic rate [101]. Accordingly, whole kidney mitochondria from rats exposed to 48 h of 0.25 M NH_4_Cl in the drinking water showed faster calcium uptake and higher resting respiration [5]. On the other hand, lipid accumulation in opossum kidney cells (OKP) cells, a model of renal proximal tubule cells, inhibits ammonium secretion, a process that is similarly observed in Zucker diabetic fatty (ZDF) rats, a model of type 2 diabetes [13, 14]. Therefore, acid–base status directly influences proximal tubular glutamine metabolism with impact on gluconeogenesis and lipid metabolism, while impaired lipid metabolism or gluconeogenesis impacts renal capacity of excreting acids.

Two central questions derive from these observations: (1) Does deranged metabolism define trajectories towards faster or slower kidney function decline in chronic kidney disease (or recovery vs. declining kidney function in an AKI to CKD scenario) or does it simply reflect disturbance from other causes? Cippà et al. identified early markers associated with these trajectories in biopsies of patients submitted to renal ischemia and reperfusion because of transplantation [29]. They identified that genes associated with mitochondrial function, senescence, and inflammation were among the most prevalent genes associated with different trajectories. (2) Given the roles of pH in metabolism and mitochondrial function described previously here, is pH a key factor influencing these trajectories, or is it only a sensitive biomarker of kidney damage?

### Cell energy metabolism beyond the proximal tubule

It is evident from the discussion above that our knowledge in renal energy metabolism is highly centered on the proximal tubule metabolism. However, other segments are also essential for the acid–base balance, and further research is necessary to shed light on the role of cell metabolism in the whole nephron in health and disease. Intercalated cells are an intriguing case for the study of renal cell energy metabolism. While animal cells are energized by Na^+^/K^+^ ATPase activity, there are strong evidences that intercalated cells do not express this protein and are rather energized by a H^+^-ATPase [25]. Intercalated cells display a sizeable Golgi apparatus and are also known as mitochondria-rich cells, a term that comes from the higher proportion of mitochondria in comparison to principal cells [28, 60]. Type A intercalated cells accumulate mitochondria in the apical cell pole and do not present the typical enrichment of mitochondria next to the basolateral membrane [28, 61]. Renal epithelial cells in the cortex rely mostly on oxidative phosphorylation to generate ATP, but type A intercalated cells have a high anaerobic glycolytic capacity, which may produce the driving force for H^+^ secretion [10, 42, 116]. Urinary acidification capacity is mostly preserved in CKD [75, 102], but it is compromised in renal tubular acidosis. Whether tubulointerstitial injury and distal RTA impose or are associated with differential metabolic demands on type A intercalated cells is still an open question. Given the potential role of the thick ascending limb in urine acidification [33], these studies should also cover the loop of Henle.

### How would alkali therapy protect renal metabolism?

Metabolic acidosis in CKD is a consequence of nephron loss and reduced ammoniagenesis. With less functional units, kidneys lose capacity for generating new bicarbonate and slowly lose the battle against daily metabolic acidification. Given that the remaining functional nephrons must deal with multiple tasks other than acid–base balance, we hypothesize that kidneys may need to sacrifice efficiency in certain functions to keep homeostasis of multiple parameters at acceptable levels. There are hints supporting this hypothesis. NRF2 is a ubiquitously expressed master regulator of oxidative stress, with roles in intermediary metabolism and mitochondrial function [49, 65]. Mouse deficient for NRF2 (*Nfe2l2*) have strongly downregulated expression of SNAT3, the main importer of glutamine in the proximal tubule and a crucial player in the response to metabolic acidosis and ammoniagenesis [72]. To our surprise, acid-loaded *Nfe2l2* knockout mice showed the same severity of metabolic acidosis in comparison with wild-type mice under the same conditions. However, after a week under acid-loaded conditions, *Nfe2l2*-deficient mice show elevated markers of kidney injury and oxidative stress despite a similar grade of acidosis in relation to wild-type mice [72]. Potentially, with cells trying to cover too many tasks at the same time, some slowly fall behind. Alleviating acid–base stress could therefore be a way of releasing the pressure on one of the multiple tasks that a cell has to handle in pathological conditions. Parallel scenarios can be also observed in other contexts. For example, metabolomic analysis has shown that acidosis induces cellular metabolism reprogramming of solid tumors via NRF2 [67]. Authors observed that intermediate metabolites are redirected away from other important metabolic processes in solid tumors during acidosis. However, additional investigation is necessary to demonstrate that acidosis exacerbates metabolic stress in kidney disease.

### Early markers of eubicarbonatemic metabolic acidosis

Chronic kidney disease leading to eubicarbonatemic acidosis is a plausible hypothesis for at least two reasons. First, why would kidneys undergoing reduced kidney function suddenly fail to control acid–base balance if not in a slow and undetectable fashion until systemic markers change beyond the range of normality? Second, acid–base disorders are primarily determined by blood gas analysis, which is an exam using blood as material for investigation. Blood is not an isolated solution in a hermetically confined pipe, but a solution in continuous exchange with the interstitial space and then with the cells. Where would acidosis start? This depends on the source of acidosis. If it occurs via CO_2_ intoxication or ingestion of acid, we would have an extracellular-intracellular cause. But in the case of kidney disease, if fixed acids originated from the metabolism are the acidifying factor and kidneys are not capable of adjusting to this daily challenge, the origin of the acidosis comes from the cells and therefore occurs in an intracellular-extracellular fashion (i.e., from the net endogenous acid production). Acids generated in the intracellular space would not only meet intracellular buffers but would also move to the interstitium meeting the next layer of buffers. The effect in the bloodstream could only be visible after several layers of protection fail. Therefore, the closer we look at the origin of the event, the earlier we could detect the derangement (a counter-argument against this potentially reductionist approach would be the loss of information because of emergent properties, but it does not seem plausible in this case given that this is just a shift in focus within the same level and scale [86]). Urine citrate excretion has been proposed as an early marker of acid retention [45]. Citrate is a key factor in cell energy metabolism participating in the citric acid cycle or Krebs cycle. The metabolization of citrate yields a net gain of bicarbonate. The principle is that an organism undergoing acid retention would reabsorb more citrate, and less citrate would appear in the urine. The strategy of measuring urine citrate goes along with what is proposed here: that cell energy metabolism is essential to understand acid–base disorders and that the closer one surveys the origin of the event, the earlier it could be detected. The problem is that citrate is not only affected by acid–base conditions, but also by multiple other metabolic requirements of the cell. Further demonstration that it could be a useful marker has been published [43]. These observations must be expanded, and a panel of metabolites representing accurately the acid–base condition of the cell might substitute this or other single markers in the future.

## Additional mechanisms

A series of additional mechanisms has been proposed and most probably plays relevant roles in the outcomes of acute kidney injury, acute kidney disease, and chronic kidney disease. We briefly explore some of these mechanisms below.

### Klotho

Klotho functions as a co-receptor of fibroblast growth factor 23 (FGF23) and mediates phosphate excretion, the main titratable acid. Additionally, it has renoprotective effects and regulates inflammation [54]. It exists both as a membrane-bound or soluble molecule. High pH activates the calcium-sensing receptor (CaSR) in the distal convoluted tubule which activates a disintegrin named metalloproteinase 10 (ADAM10) [141]. The disintegrin cleaves membrane-bound klotho, generating soluble α-klotho. Low pH has opposite effects. Patients with CKD showed reduced α-klotho levels early in the disease, and alkali therapy increased excretion of α-klotho in a pilot study with patients with CKD [46]. Therefore, it is tempting to speculate that alkali therapy protects kidney function also via protection of klotho levels. Mice subjected to a crystal nephropathy model and treated with alkali therapy show preserved renal α-klotho levels despite severe tubulointerstitial injury [90].

### pH sensing

The proper response of the kidneys and lungs to acid–base challenges relies on precise pH sensing by the kidneys and peripheral and central chemoreceptors. Kidneys express a large array of proteins in which H^+^ functions as an allosteric modulator or a ligand. They are ionic channels, enzymes, and G protein-coupled receptors (GPCRs). Some examples are TWIK-related acid-sensitive K + channel (TASK) [20], acid-sensing ion channels (ASICs) [24], insulin receptor–related receptor (IRRR) [34], soluble adenylyl cyclase (sAC) [98], ovarian cancer G protein-coupled receptor 1 (OGR1/*Gpr68*), G protein-coupled receptor 4 (GPR4), and T cell death-associated gene 8 (TDAG8/*Gpr65*) [57]. Moreover, an intracellular pH-senstive proline-rich tyrosine kinase 2 (PYK2) and a bicarbonate/CO_2_ sensing protein receptor protein tyrosine phosphatase (RPTPgamma) have also been identified [15, 69]. Disruption of several of these sensing mechanisms leads to poor management of acid–base balance by the kidneys or to insensitivity of mechanisms regulated by acid–base conditions. For example, TASK2 knockout mice display a phenotype similar to human proximal renal tubular acidosis [126], and OGR1 knockout mice display poor coordination between urinary acidification and calcium excretion [58]. GPR4 deficiency in mice fully blunts acid-dependent proliferation of type A intercalated cells and induction of transporters involved in acid–base balance in these cells [27]. These animals also show lower excretion of titratable acids and lower ammonium excretion in response to acid load [117, 118]. However, this might be indirectly caused by the respiratory acidosis of central origin observed in these animals [27, 66]. The role of pH sensing is also extended to kidney injury conditions, as seen in the inhibition of ASIC1a with psalmotoxin 1 (PcTx1) in mice, which attenuated injury caused by renal ischemia reperfusion [109]. PcTx1 not only increases the affinity of ASIC1a by H^+^, but also functions as an agonist of ASIC1b in neurons [26]. GPR4-deficient mice are also protected from renal ischemia reperfusion injury [35]. In summary, pH sensing mechanisms play central roles in acid–base balance in health and in pathological processes. However, despite extensive research on either the pH-sensing properties of these proteins or on their role in disease, there are only few studies that reported a systematic investigation of how both aspects interact. There is plenty of room for research in this direction.

### Acid–base and immune responses

The interaction between acid–base conditions and immune responses has been explored by research in several fields, such as oncology, pain and nociception, pulmonology, and gastroenterology, but it has been understudied by renal physiologists and nephrologists [31, 56, 57, 91, 143]. Acid–base conditions influence differentiation and motility of immune cells and their capacity to release substances [38, 97, 142]. Given the increasing attention towards the role of inflammation in kidney diseases, it is expected that this gap will be narrowed in the next few years. In the previous sections, we explored some examples of this interaction, such as ammonium as a trigger of the alternative complement pathway and acidosis-stimulated chronic activation of hormones driving inflammation of the renal tissue. The effect of acidosis in the immune cell activity in kidney disease is mostly unknown and deserves special attention. Recently, we showed that CD4^+^ T cells and inflammatory monocyte levels were reduced in kidneys of mice subjected to a crystal nephropthy model and receiving oral bicarbonate [90]. However, alkali therapy could also have extrarenal effects with relevance to the kidneys. A hypertensive kidney disease rat model (Dahl salt-sensitive) under oral bicarbonate intake displayed splenic and renal macrophage polarization towards an anti-inflammatory M2 type suggesting that alkalinization impacts pre-renal differentiation of immune cells with impact in the kidneys [96]. Interestingly, administration of esomeprazole, a proton pump inhibitor, blunted the anti-inflammatory effect of NaHCO_3_ intake in rats. Authors proposed a mechanism that gastric acidification is directly or indirectly sensed by mesothelial cells of the peritoneum which sends an anti-inflammatory message via cholinergic signals to the spleen [96]. This novel mechanism would at least partially bypass canonical acid–base sensing systems like peripheral and central chemoreceptors. However, its actual impact in kidney function has yet to be demonstrated. Oral bicarbonate could also influence kidney disease through the relationship between intestinal dysbiosis and mitochondrial dysfunction in CKD [76]. Concerns with ocean acidification have prompted multiple studies aimed at identifying whether lower pH could impact the diversity of microorganisms in the sea. Hypercapnia affected the intestinal microbiota of fish and crab [41, 73]. Likewise, there is a clear impact of ruminal acidification on the microbiota of different species of cattle [53, 70]. Acidic water has been shown to affect intestinal microbiota of mice [107, 139], but results are contradictory and have not been reproduced by others [144]. Moreover, the single study performed in humans did not find any impact of acidic water in the intestinal microbiota of young males [47].

## Expanded conceptual framework integrating interactions between pH homeostasis and progression of chronic kidney disease

**Fig. 3:**
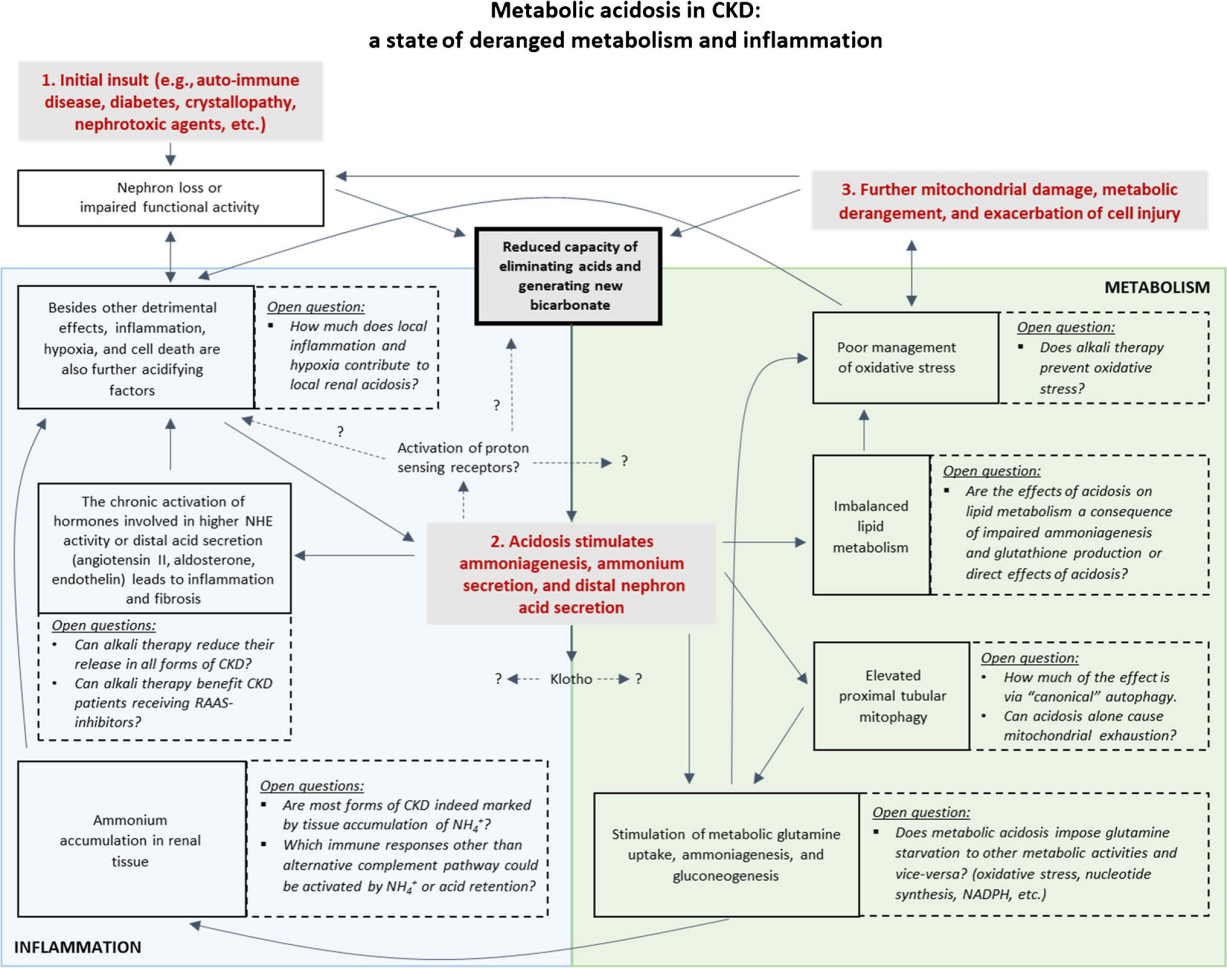
Conceptual framework how chronic kidney disease, inflammation, and deranged metabolism form a vicious cycle involving metabolic acidosis as an engine. Nephron loss and impaired renal function reduce kidney capacity of eliminating acids and generating new bicarbonate which leads to accumulation of acids in the organism. Renal responses to acidosis exacerbate inflammation and deranged metabolism that ultimately reduce kidney function and kidney capacity of keeping pH homeostasis. Steps of this network are shown in continuous black boxes, and open questions related to each of these steps are shown next to it in dashed black boxes. Inflammation and metabolism domains are artificially delimited in different colors as some of these steps may belong to both domains

Most probably, certain effects of pH on kidney function act independently of each other. However, independent effectors may synergize into further kidney injury, and some others may actually be dependent on each other. Here, we propose a conceptual framework with multiple known factors involved in acid–base-dependent progression of chronic kidney disease and how they would evolve from early to late stages of CKD (Fig. 3). In this figure, we also list key open questions related to multiple steps of this framework.

## Concluding remarks

Extensive research in the XX century characterized how metabolic activities of all cells generate the input of the acid–base balance and how mainly the kidneys and lungs control the output. Dietary habits influence net production of acids and bases and are important factors determining the daily stress to pH homeostasis. Recent research has identified that metabolism and inflammation are central pathways to multiple forms of kidney disease, and there is evidence that acid–base status is a potent modulator of these pathways. Models proposed in the past couple of decades on how acidosis impairs kidney function in chronic kidney disease and how alkali therapy protects kidney function did not include the role of cell metabolism (and potentially immunometabolism) as core pieces. H^+^ has pleiotropic effects in biological systems, and it is believed to affect organisms from multiple angles. It is time to revisit relevant knowledge acquired since the late XIX century and further elaborate a holistic framework that includes this diversity. However, certain factors might have a more dominant effect than others, and identifying them may be a powerful tool to manipulate pathways involved in the progression of chronic kidney disease. We propose that cell metabolism is at the core of the pH-dependent events associated with the progression of chronic kidney disease. If this is the case, a deep understanding of how pH affects biochemical reactions in a systemic fashion may provide a powerful tool to control the progression of renal and extrarenal chronic diseases with deranged metabolism.
